# Determinants of Household Food Basket Composition: A Systematic Review

**DOI:** 10.18502/ijph.v49i10.4681

**Published:** 2020-10

**Authors:** Seyyed Reza SOBHANI, Mina BABASHAHI

**Affiliations:** 1. Department of Nutrition, School of Medicine, Mashhad University of Medical Sciences, Mashhad, Iran; 2. Department of Community Nutrition, School of Nutrition Sciences and Food Technology, Shahid Beheshti University of Medical Sciences, Tehran, Iran

**Keywords:** Socioeconomic factors, Household, Food

## Abstract

**Background:**

Demographic, socioeconomic, and environmental determinants are important to population health status in all countries and diet is the main way that these factors could affect health. We aimed to conduct a systematic review of recent research evidence about these determinants of household food basket composition.

**Methods:**

The PRISMA guideline was used to the reproducibility of this systematic review. Three databases including PubMed, Scopus and Google Scholar were systematically searched from 1991 to Dec 2017.

**Results:**

Thirty four studies were included. Most studies were done in the United States. Three categories of determinants including the demographic, socioeconomic, and environmental define the contribution of different food groups in the household food basket. These factors determine the healthiness of family diet.

**Conclusion:**

Many determinants affect household food basket. Comprehensive consideration of policymakers to these factors is essential to creating and maintaining a healthy society.

## Introduction

Socioeconomic factors are major determinants of health in high, middle, and low-income countries and diet are one of the main ways that socioeconomic factors can affect the health (1, 2). The relationship between Socioeconomic Status (SES) and diet has been studied mostly based on food choices and nutrient intake (3). SES is represented by multiple indicators including income, education, and occupation, all of which may operate independently or interact in leading to inequalities that influence food choices (4). Multiple food-related choices that are made every day are linked to complex interactions among economic, culture, social class, or food environment (5). Secure nutrition addresses not only the required level of calorie intake but also the proper balance of food items in households’ food basket (6). Food shopping behaviors and the household food purchases pattern influence food available in the home and individual intake through simple availability and from social influences (7–9). Investigative food purchase patterns at the household level may provide possible reasons for less healthful individual food intake (9). For instance, purchasing and consumption of unhealthy diets, in particular, eating fewer fruits and vegetables, is strongly patterned by SES (10). Potential socioeconomic determinant on purchasing decisions and family food basket composition are income/expenditure, expenditure patterns, prices, market access, and household characteristics (11, 12).

Since making dietary decisions are in relation to food and not nutrients and food choice differences between socio-economic groups lead to differences in nutrient intake better understand socioeconomic factors that influence the household food basket (HFB) is important (13–15). As well as, designing dietary interventions in public health policy will benefit from a research focus on socioeconomic determinants of HFB (5).

Therefore, we aimed to investigate the socioeconomic determinants of household food basket composition by systematically reviewing the evidence.

## Methods

### Conceptual Framework

**Fig. 1:**
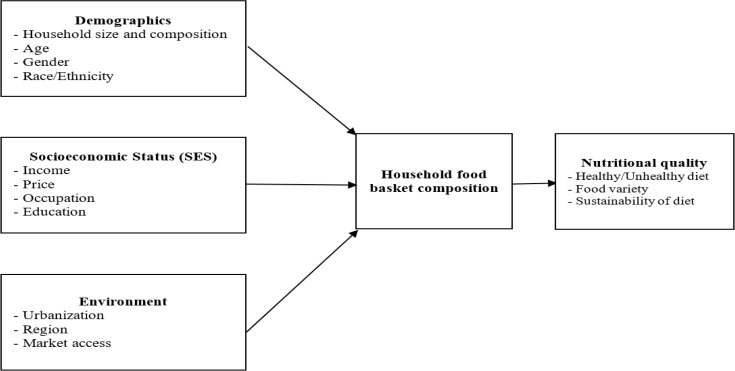
Conceptual framework of household food basket composition determinants

In this study, a combination of two conceptual frameworks were used. First, one was the conceptual framework of food insecurity and its relation to overweight that was developed (16). According to this model, food insecurity influences overweight directly as well as indirectly through lifestyle factors. Furthermore, food insecurity is influenced by two demographic variables (age and ethnicity), three socioeconomic variables (education, income and occupation), two government assistance variables (welfare status and food stamps), three environmental variables (household size, urbanization and region of country) and five lifestyle variables (vigorous exercise, television time, percentage of dietary energy as fat, percentage of dietary energy as saturated fat and total energy intake). Second framework was a conceptual model of the components in the food choice process developed (17). This model represents three types of factors (life course, influences, and personal system) and the process involved in a single choice event. The model includes five major categories of influences upon food choice: Ideals, personal factors, resources, social framework, and food context. By using these two conceptual frameworks, the conceptual framework of household food basket composition determinants was developed and used in the present study (Fig. 1). Three categories of factors including demographic, socioeconomic, and environmental factors affect the contribution of different food groups in the family food basket. Through this way, these factors determine the quality and quantity of household diet.

## Methods

In order to the reproducibility of systematic review present study, its methods and results are reported according to the PRISMA guideline (18). Three databases including PubMed, Scopus and Google Scholar were systematically searched from 1991 to Dec 2017. Articles in English were considered. We applied the same search strategy in all electronic databases. Key terms were categorized in three groups and used in combination with each other as follows:

(Famil^*^ OR Household^*^) AND (“food purchase*” OR “food choice*” OR “food basket”) AND (“Socioeconomic status” OR Income OR Employment OR Education OR Occupations OR “Ethnic Group*” OR “Social Environment” OR “Social Class” OR “Social Conditions” OR Sex OR “Age Group*” OR gender OR age OR “Socioeconomic Factors*” OR Ethnic OR race).

After removing duplicates choosing related studies to socioeconomic determinants of household food basket composition conducted orderly by screening the titles, reading abstracts and then reading full texts. Choosing final related studies conducted by two independent reviewers and disagreements were resolved by consensus. Studies that focused only on children, parents, special age groups (e.g. elderly) and did not include whole household excluded. If food basket assessment includes just one food group or did not consider the composition of that, were also excluded. Studies without English full text were removed. The reference lists of selected studies were searched in order to find any studies that not included by the electronic search. This process added two new studies.

For each study, the following data were extracted: study’s country, year, sample size, food basket assessment method, measures of SES and the main outcome or conclusion of the study were summarized in data extraction table. Because each study was designed to address different outcomes and associations, we reported associations adjusted for the relevant socioeconomic determinants.

The quality of eligible studies was evaluated using STROBE checklist. This checklist includes 22 items was constructed in order to assess the quality of observational studies. The quality assessment was done independently by two authors and consultation of the third reviewer in the event of a discrepancy.

## Results

**Fig. 2:**
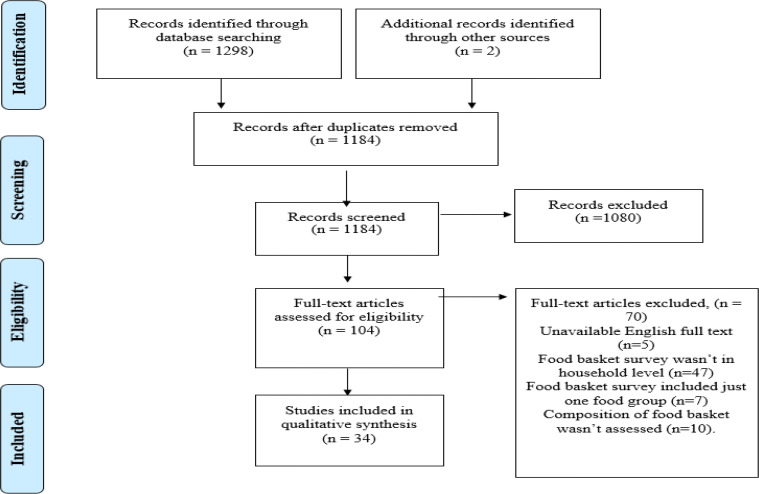
PRISMA diagram

As shown in the PRISMA diagram of the present study (Fig. 2), from 1182 first search results which irrelevant 1080 irrelevant of them were removed in screening steps, 104 full texts were assessed. Finally, 34 studies were included in the review that is shown in Table 1. In addition, further information about the included studies will be available by contacting corresponding author of this systematic review. Although studies from the United States (n=10) made up the biggest share, studies from different parts of the world (Europe=10, Australia=5, Canada=4, Asia=2, Brazil= 2, Africa= 1) were included too.

**Table 1:** Studies investigating the association between socioeconomic factors and household food basket composition

***Reference number***	***Year of data collection***	***Sample size (household)***	***Nation***	***The measure of Household Food Basket***	***Measure of SES***	***Outcome/Conclusion***
11	2014	80	Romania	Buying behaviors	Income	Self-consumption behavior was seen in urban low-income families and fresh and healthy foods basis behavior were in higher income families
49	1996–97	882	USA	Seven-day food report	Prices, food access	Prices are significant determinants of food purchases, but food access is not.
21	2001–2009	5020	Scotland	14-day record of purchased food	The Scottish Index of Multiple Deprivation	In deprived households, more of healthy food were lowest consumption, while total bread was highest consumption.
50	2000–2008	-------	Slovak republic	food expenditures	Income	There is no significant influence of the household income differentiation on the purchases of the particular food groups.
10	2010	24,879	UK	Purchases of (1) fruit and vegetables and (2) less-healthy foods/beverages	Head-of-household occupation	Higher occupational social class was significantly associated with greater food expenditure and healthier purchasing
41	2001–2009	550	Scotland	14-day record of purchased food	The Scottish Index of Multiple Deprivation	Single-parent household and living in the most deprived areas were associated with higher mean energy density
24	1999	7,195	USA	Reported purchasing from milk, bread, cereal, and soft drinks food categories.	Income, age group of household heads, the nature of household heads, prices	Households with college-educated heads, higher incomes and older households made significantly healthier choices.
27	2000–2012	157,142	USA	Scan purchased goods	Race/ethnicity	Both black and Hispanic households had lower purchases of highly processed and ready-to-eat foods compared with white households
30	2002–2003	48,470	Brazil	7 day record of consumption food	Education of the head, presence children, adolescent, elderly persons, income	The rice and beans pattern was associated with the presence of adolescents in households. A mixed pattern was associated with a higher income and education.
51	1998–2008	-	Slovakia	Input empirical data on net incomes and expenditures for the household food	Income distribution	The demand for potatoes and vegetables is elastic in the households with the lowest incomes
19	2000	529	Australia	Respondents indicated households’ usual choice of 16 staple grocery foods	Income, household size	As household size increased, grocery purchasing behavior was observed to be less consistent with dietary guideline recommendations
4	2001	2000	Belgium	Record purchases by home scanners	income, Family size, Number of children, Region of living, Education, Profession, Age	The poorer people spend significantly less money on food in general and specifically less on fish, dairy products and vegetables.
37	2004–2005	334	Fiji	Food frequency questionnaire (FFQ)	Rural and urban areas, parental skills and knowledge- based abilities, number of children, education	Urban high-embodied-capital households spend significantly more on food purchases especially processed foods than do urban low-embodied-capital or rural households.
2	2000	1003	Australia	interview	Education, occupation, household income	The least educated, those employed in manual occupations and residents of low-income households purchased fewer healthy food.
31	1997–1998	1998	USA	Household food purchase data	Income	Low-income households purchased 3.3 percent fewer fruits and vegetables (by weight) per person than high-income households.
34	1996	9793	Canada	1-week food expenditure data	Income	Low incomes household had low access to milk products and fruits and vegetables.
23	2008–2009	4,412	Netherlands	Daily register all purchases on a home scanner during a twenty-week period	Gender, Education, Age, Income, Region	People with higher education and those living in a more urban area are more likely to purchase sustainable products.
35	2010	24,879	UK	Purchase records of households over 52 weeks	Types of supermarkets (high- or low-price supermarkets)	Households using low-price supermarkets purchased significantly higher percentages of energy from less-healthy foods.
22	1996	10924	Canada	Family Food Expenditure	Household size, composition, income, education	Household socio-demographic characteristics have a strong influence on vegetables and fruit purchasing.
28	2000–2013	164,315	USA	The U.S. packaged food purchases	Race, ethnicity	Disparities in food purchases shrank over time by race/ethnicity but not by income level.
32	1989–2006	2441	Portugal	Self-registration diary of food items	education, household urbanization degree, location the household per capita income	The simultaneous effects of socio-demographic variables and time were significant for all food groups
12	2012	201	Iran	questionnaire	Sex, Age, Income, Education, Household size	Older respondents, females, households with high income and high educational levels were more likely to choose healthy foods.
52	2004	115	USA	interviews	Highest household education, race, ethnicity	Race and ethnicity were very effective on food choices
45	2001–2002	1708	Canada	questionnaire	Household education, income, employment	Lower levels of the 4 SES factors contribute to poorer food intakes
26	2015	114	Australia	questionnaire	Generation, role, ethnic	The women in each generation influenced on fruit and vegetable intake.
38	2003	2564	Australia	Compliance with dietary guideline recommendations	Education, occupation and household income.	Area SES was associated with some food purchasing behaviours independent of individual-level factors.
25	1995–96	15065	Brazil	A one-week survey	Household age/gender composition	A significant shift in the distribution of per capita food distributions when comparing member count versus adult equivalent-based per capita distributions.
53	2007–2008	90	USA	Annotated food purchase receipts were collected for a four-week period	Income	Higher-income households spent more money on both healthy and less healthy foods.
33	1987–88 and 2004–05	97763	India	National Sample Survey Organization (NSSO) data on consumption expenditure	Rural and urban sectors, geographical regions, income	Food consumption pattern of Indian households was found to be in conformity with Engel and Bennett’s’ law of consumption.
46	1986–2001	35048	Canada	the family food expenditure survey	income	Significant positive relationships between income and most nutrients.
39	2003	2564	Australia	questionnaire	Financial and physical barriers for shopping.	financial and physical barriers were more likely effected purchase fast foods
54	1998	105	USA	Food purchase receipt data for at least 6 weeks	size, composition, use of home- grown foods, education, occupation, income, ethnicity, gender, age	Poor nutrition quality of purchases were in families with lower socioeconomic status, more children, and younger age of the primary shopper.
55	2012	1581	USA	The USDA’s National House-hold Food Acquisition and Purchase Survey	Neighborhood food store availability	Existence of supermarket among households was influenced in purchas-ing water and low-calorie beverages and fruits and vegetables.
20	1987–88	4,273	USA	Household food consumption survey	Income, education level, race, urbanization, region, house-hold size and composition.	Higher education was associated with spending less of the food budget on meat and more on vegetables

### Demographic factors

#### Household Size and Composition

Household size was an important determinant of expenditures on food, consistent with analyses of household food expenditures in different countries. As Australian household size, increased, grocery-purchasing behavior was observed to be less consistent with dietary guideline recommendations (19). In addition, larger US households allocate more of their food expenditure to beef and pork and less to bread and juice (20). In the case of household composition, single-parent Scottish household had the highest mean energy density of all the household surveyed (21).

#### Age

Among Canadian households, households with older adults spent a greater share of their income on vegetables and fruit, whereas households with children purchased a greater quantity of milk products (22). In another study, older people are more likely to purchase sustainable food products than younger people (23).The nutritional quality of bread and cereal purchases increased sharply with age of household head. Moreover, older households much more likely to substitute diet for regular soft drinks (24). In addition, households with more children and teenage members tended to spend more of their food budgets on dairy products (20, 25).

#### Gender

In Mashhad (northeast of Iran), older respondents and females were more carefully for health than young respondents and males (12). The women in each generation influenced fruit and vegetable intake by controlling purchasing decisions, insisting on consumption, monitoring and reminding, utilizing food as a prerequisite for conditional treats, instigating and enforcing food rules, and restricting others’ food choices (26).

#### Race/Ethic

In one study, compared with white households, both black and Hispanic households had lower purchases of highly processed and “ready to eat” foods (27). Another investigation of changes in food basket composition during 2000–2013 shows that although Hispanics and non-Hispanic others had the highest energy and sodium density in 2000; these groups show the largest declines in energy and sodium density 2013. Non-Hispanic black households had the highest values for energy, sugar, and sodium density, which persisted across time (28). In addition, black households allocated more of their food budgets for pork, poultry, other meat, fish, eggs, and juice than White households did, but less for dairy, bread, and fruits. Non-Hispanic white households tended to pay more for poultry, other meat, bread, and juice than other households (20).

### SES factors

#### Income

Among Romanian family, the high-income families were changing their consumption patterns being more oriented to healthy and organic foods (11). With the growing income among Slovak households, the expenditure share on starchy foodstuffs group that prices of this kind of food group were quite low declined in the total household expenditures on food. The share of expenditures on the group of fruit and vegetables has a rising tendency corresponding with the households’ incomes (29). In Brazil, a mixed pattern including healthy and unhealthy food was associated with a higher income and education (30). The report of comparison food purchases by U.S households among different income levels find that low-income households purchased 3.3 percent fewer fruits and vegetables (by weight) per person than high-income households (31). Portuguese households with larger incomes had higher relative contributions in the household food availability from fruits, meat/meat products, fish/seafood, vegetables, and nuts. Potatoes, cereals and sugar/sugar products were negatively correlated with income (32). In India in case of cereals, pulses, edible oils, and vegetables, the expenditure share reduced with the increase in income, while for growth in the consumption of high value agricultural (milk and milk products, non-vegetarian products and fruits) commodities with the rise in income (33). Another study in Canada shows that low-income households purchased significantly fewer servings milk products, fruits, and vegetables than did higher-income households (34).

#### Price

When U.S household product choices within four important grocery categories including milk, bread, breakfast cereals, and soft drinks were investigated, price differences across varieties were small or nonexistent, so that higher cost was a barrier limiting access to healthier products (24). However, an observational panel data on purchases of fruit and vegetables and less-healthy foods/beverages in UK shows that low-price supermarkets purchased significantly lower percentages of energy from fruit and vegetables and higher percentages of energy from less-healthy foods/beverages than households using high-price supermarkets (35). In a study that considered the roles of prices and food access simultaneously, prices were significant determinants of food purchases, but supermarket access had limited influence allocation (36).

#### Occupation

Among UK, household higher occupational social class was significantly associated with greater food expenditure, which was in turn associated with healthier purchasing (10).

#### Education

Previous studies have shown that people’s education levels can influence household food choices. For example, in Brazil (30), Canada (22), Portugal (32), Mashhad (northeast of Iran) (12), USA (20), and Netherland (23) have been revealed that people with higher education as compared to those with lower education level were more likely to purchase healthier and sustainable products.

### Environment factors

#### Urbanization

The 2002–2003 Brazilian Household Budget Survey shows that there was no difference in dietary availability patterns between urban and rural areas (30). But in Fiji, findings indicated that urban high-embodied-capital households speeded significantly more on food purchases, purchased a greater proportion of processed foods, and had children with higher body mass indexes (BMI) compared with urban low-embodied-capital or rural households. Parental embodied capital was a measure of parental skills and knowledge-based abilities in urban environments (37). Portuguese households located in urban areas had a higher contribution of milk/milk products, fruits, non-alcoholic beverages and fish/seafood; while at semi-urban areas there was a higher contribution of alcoholic beverages and at rural areas higher values for the other food groups, except for meat/met products a medium effect size was found (32). The consumption of cereals was comparatively higher in the rural sector in all the regions of India compared with the urban sector (33).

#### Region

A study in Australia shows that individuals living in the most deprived areas had a higher mean energy density than those living in the least deprived areas (21). In Melbourne, residents of low-SES areas were significantly less likely than their counterparts in advantaged areas to purchase grocery foods that were high in fiber and low in fat, salt, and sugar; and they purchased a smaller variety of fruits. Low SEP is less likely to buy grocery foods that accord with diet-related health promotion messages and dietary guidelines and had significantly higher odds of purchasing a lower variety of fruits and vegetables (38). According to Portuguese regions, differences were found between the Portuguese regions for all food groups, except for sugar/sugar products (32). There was a sharp contrast in food preferences in different regions for diary and non-vegetarian products by different groups in India (33). A study in the USA showed significant regional and seasonal differences in food budget allocation. For example, households in the west devoted a greater proportion of their food budget to dairy products and fruits than other households and less to pork and other meat (20).

#### Market access

Among U.S households, those who traveled more than 20 min to a supermarket devoted less of their food expenditures to non-canned fruit and vegetables than those who had shorter travel time (36). In another study, more-frequent trips and fewer small trips were associated with healthier purchasing (35). In Australia, householders experiencing financial and physical barriers were more likely to purchase frequently chain fast foods. While limited access to a car resulted in a lower likelihood that the nutritious options were purchased for two core food items (bread and milk). Limited evidence was also found that reduced vehicle access might be associated with less frequent purchasing of some nutritious food items (39).

## Discussion

The present systematic review showed that three categories of factors including the demographic, socioeconomic, and environmental effect on the contribution of different food groups in the family food basket. Consequently, these factors determine the healthiness of household diet.

Household’s size and composition, age, gender, and rice/ethics are demographics factors that studies investigated their association with the composition of the household food basket. As family size increase, adoption of household purchase pattern with the nutritional recommendation and the share of vegetable and fruits decrease. Larger households prefer buying larger quantities that often cost less per unit (40). Lower food spending among larger households may also be because of their tendency to substitute less-expensive foods with lower nutritional quality. About family composition, the dominant role of children in food choice leads to the higher energy density in single parent families (41). Households with younger age composition are at highest risk for poor nutrition quality purchase. Health concerns in older adults lead to more emphasis on purchasing vegetables and fruit (42). In the case of gender, female shifts household purchases to higher healthy types of food. Race/ethnicity can effect on family food choices. Different race in the USA has a different composition of food purchase. Choices of Black and Hispanic household are less healthy compared with American. In reviewed studies, socioeconomic factors are income, price, occupation, education or an index that is a combination of them. Generally, higher income households are more likely to choose healthy foods. Increasing income is associated with better nutrition. Higher-income gives people more freedom in food choices (43). The higher price of healthy food leads to unhealthy choices among lower-income families. Moreover, higher occupational social class is associated with healthier purchasing. In the case of education, there is a positive effect of education on the purchase of vegetables and fruit. Higher education individuals are more aware of diet–disease and more likely to believe that their food choices can influence their health (44). In addition to the potential effect of education level on awareness of healthy habits, it has an influence on jobs position and their resulting income (45). Overall, a household with lower socioeconomic status are less likely to purchase foods consistent with nutritional recommendations. Higher SES groups compared with lower SES groups receive more health messages and they have a greater ability to take action on nutritional recommendations and less resistance to change (46). Totally, household size composition, education, and income combined explained only 21% to 29% of the variation in food purchasing (22).

Urbanization, region, and market access are environmental affected factors on family food choice. In developed countries like the Netherlands and Portugal, living in an urban area is associated with healthier and sustainable food purchase. While in developing countries (e.g. Fiji), urban households purchase more processed foods. The region may be differentiated based on food availability, accessibility, and affordability, making the purchase of some types of foods more difficult in disadvantaged areas (38). The residents of low-SES areas are less likely to buy healthy foods such as fruits and vegetables. In the case of market access, more-frequent trips and fewer small trips are associated with healthier purchasing. Distance to main roads and time to market are common indicators of how accessible rural markets are and reflect the unobserved transportation and transactions costs (47, 48). Householders with physical limitations purchase more fast foods because they cannot carry groceries to or prepare food at home easily (39).

## Conclusion

Nowadays, the growing trend of diseases, especially non-communicable diseases, multiplies the need for preventive measures against these diseases. One of the most important ways to achieve this goal is to maintain a healthy family food basket. As this study stated, demographic, socioeconomic, and environmental determinants are highly influential on the health of this basket. Public health policies should be dedicated to the optimal use of these factors to achieve a healthy and active community.

## Ethical considerations

Ethical issues (Including plagiarism, informed consent, misconduct, data fabrication and/or falsification, double publication and/or submission, redundancy, etc.) have been completely observed by the authors.
